# Immune DNA methylation in depression: cross-sectional and longitudinal study

**DOI:** 10.1192/bjo.2025.10065

**Published:** 2025-07-10

**Authors:** Marisol Herrera-Rivero, Matthias Nauck, Klaus Berger, Bernhard T. Baune

**Affiliations:** Department of Psychiatry, University of Münster, Münster, Germany; Joint Institute for Individualisation in a Changing Environment (JICE), University of Münster and Bielefeld University, Münster, Germany; Institute of Epidemiology and Social Medicine, University of Münster, Münster, Germany; Institute of Clinical Chemistry and Laboratory Medicine, University Medicine Greifswald, Greifswald, Germany; German Centre for Cardiovascular Research (DZHK), Partner Site Greifswald, University Medicine Greifswald, Greifswald, Germany; Department of Psychiatry, Melbourne Medical School, The University of Melbourne, Parkville, Australia; The Florey Institute of Neuroscience and Mental Health, The University of Melbourne, Parkville, Australia

**Keywords:** Depression, diabetes, DNA methylation, immunity, inflammation

## Abstract

**Background:**

Immune dysregulation contributes to the pathophysiology of depression and is a potential link between depression and comorbid medical conditions. DNA methylation is a dynamic transcriptional regulator of the immune system.

**Aims:**

To study changes in DNA methylation of disease- and comorbidity-associated immune genes in patients with and without depression diagnoses from the German BiDirect Study.

**Method:**

We performed a cross-sectional (baseline, y0) and longitudinal (consecutive assessments at 3-year intervals, y0, y3, y6) differential methylation analyses of 382 immune-related genes associated with depression, obesity, diabetes and/or gout in 276 patients with depression and in 207 individuals without a lifetime depression diagnosis from the BiDirect Study. In addition, we applied unsupervised clustering to identify subgroups of individuals with depression based on longitudinal methylation patterns.

**Results:**

There were no significant methylation changes between individuals with depression and controls at baseline. Follow-up analyses used to assess the top (*P* < 0.05) 151 methylation probes longitudinally identified 42 CpG sites that showed time-dependent changes associated with depression, and defined 3 depression clusters with differential profiles of serum inflammation markers at baseline. The implicated genes corresponded in the majority to those associated with diabetes risk, and were enriched in processes relevant for haematopoiesis.

**Conclusions:**

Our results suggest that immune dysregulation associated with DNA methylation profiles contributes to the pathophysiology of depression and is a plausible link to chronic medical conditions such as diabetes.

Depression is a common and disabling psychiatric disorder. Elucidating the underlying biological mechanisms has proved challenging due to the complexity of the disorder, its highly heterogeneous aetiology and symptomatology and the markedly high rates of comorbidity with other psychiatric disorders and chronic medical conditions.^1,2^ In recent years, the role of the immune system in the pathophysiology of depression has drawn increasing attention. Peripheral immunity affects mood and behaviour through the infiltration of immune factors, such as cytokines and activated T cells, into the central nervous system, where they can modulate multiple neuronal functions and neurotransmitter systems.^3,4^ Therefore, alterations in the regulatory mechanisms of the immune system contribute not only to the development and course of somatic conditions but also to those of psychiatric disorders.

## Immune dysfunction in depression and its medical comorbidities

Currently there is compelling clinical, epidemiological and genetic evidence of a broad spectrum of alterations of inflammatory and immune response processes in depressed individuals. These include changes to both innate and adaptive immunity, affecting the production of anti- and pro-inflammatory cytokines, chemokines and the relative abundance and activation of immune cell populations, among other factors, which have also been demonstrated to correlate with particular clinical features, including behavioural patterns and antidepressant response, in at least subsets of depression patients.^1,3–5^ Moreover, a clear increased risk of depression exists in the context of infection and inflammatory and autoimmune disorders, as well as in an array of long-standing medical conditions with the presence of chronic (low-grade) inflammation, including metabolic syndrome, diabetes and coronary heart disease.^4,6,7^ A relationship between immunity, depression and somatic disease is supported at the biological level by different lines of evidence, including genetic association studies^8,9^ and studies that measured immune activity through diverse methodological approaches in humans and animals.^10^ This, together with the mounting evidence showing that anti-inflammatory treatments possess antidepressant properties, supports the hypothesis that immune dysregulation links depression and medical disease.^1,5^ However, the underlying molecular mechanisms remain incompletely understood.

DNA methylation (DNAm), a reversible epigenetic modification that serves as a regulatory mechanism for cell- and tissue-specific gene expression, is a crucial modulator of the immune system. Despite being considered a relatively stable phenotype, DNAm is susceptible to the long-term effects of a number of genetic, environmental and lifestyle factors, and has been shown to play a role in human disease.^11,12^ Changes in the peripheral blood levels of DNAm in multiple immune-related genes have been reported for psychiatric and medical conditions, including depression and metabolic and immune-related diseases.^5,13–15^ We hypothesised that changes in DNAm patterns of disease-associated genes occur in subjects with depression over time and contribute to the immune profiles and increased risk of some medical conditions commonly observed in these individuals. To test our hypothesis, we investigated (a) whether immune genes associated with depression and specific medical comorbidities are differentially methylated in subjects with depression compared with non-depressed controls, and (b) how DNAm levels alter longitudinally over a 6-year time span. In addition, we explored whether the longitudinal patterns of DNAm that modulate disease-associated immunity can define immunological subtypes of depression.

## Method

### Study population

BiDirect is a prospective, observational study designed to explore the bidirectional relationship between depression and subclinical arteriosclerosis. The study design and methods have been described elsewhere.^16^ Briefly, the sample is comprised of the following: (a) depression cohort: patients hospitalised for an acute depressive episode at the time of recruitment; (b) cardiovascular cohort: patients who suffered from an acute coronary event 3 months before recruitment; and (c) population-based cohort: randomly sampled community-dwelling adults. Participants were recruited in the district of Münster, Germany, and underwent extensive phenotyping in 4 examinations over a period of 10 years, with intermediate follow-ups by self-administered questionnaires. Depression was diagnosed at recruitment using selected modules (i.e. modules A, A’, B, D and O) of the M.I.N.I. International Neuropsychiatric Interview (German version 5.0.0)^17^ to assess the presence of acute (first or recurrent) major depression with or without melancholic features, acute dysthymia, acute/former manic/hypomanic episodes or acute generalised anxiety disorder. Different measures were combined in order to confirm psychiatric diagnoses in the depression cohort. The comprehensive examination of BiDirect participants included depressive symptoms assessed using the Center for Epidemiological Studies-Depression (CES-D) scale, weight, body-mass index (BMI), diagnosis of different medical conditions, including lifetime diagnosis of depression and diabetes, information on medication use under the Anatomical Therapeutic Chemical (ATC) classification system, and the measurement of serum levels of inflammation markers, including C-reactive protein (CRP), cytokines and chemokines. A subset of 300 individuals from both the depression and population-based cohorts was selected to carry out genome-wide measurements of DNAm by array at baseline (y0) and the 2 consecutive follow-up visits taking place 3 (y3) and 6 (y6) years after baseline. Selection was performed by age, sex and participation in the first 3 examinations. The current study employed the BiDirect depression cohort as the case group. The control group consisted of a non-depressed community sample comprising individuals from the BiDirect population-based cohort without a lifetime diagnosis of depression up to y6. No other psychiatric diagnoses were considered as exclusion criteria.

The authors assert that all procedures contributing to this work comply with the ethical standards of the relevant national and institutional committees on human experimentation, and with the Helsinki Declaration of 1975 as revised in 2013. All procedures involving human subjects/patients were approved by the ethics committee of the University of Münster (no. 2009–391-f-S) and the Westphalian Chamber of Physicians in Münster, North-Rhine-Westphalia, Germany. All participants in the BiDirect Study provided written informed consent.

### Selection of comorbidities and genes

For this study, we were interested in those medical conditions that have been found to be comorbid following the onset of depression, given that it is more likely that the changes occurring in the context of depression increase the risk of developing such conditions than would be the case in the opposite scenario. To make a systematic selection, we based our choice on the recent report by Frank et al, where severe or moderately severe depression was shown to precede the onset of obesity, diabetes and gout with a relatively high (>5) hazards ratio.^18^ To select the genes for inclusion in our study, we first identified those associated with (unipolar) depression, obesity, (type 2) diabetes and gout using information collected from the Genome-Wide Association Study (GWAS) Catalog.^19^ Here, we excluded ambiguous traits (e.g. schizophrenia, bipolar disorder and depression (combined); chronic pain or major depressive disorder (pleiotropy)) and those related to other disorders (e.g. depression in multiple sclerosis), as well as associations arising from trait and single-nucleotide polymorphism (SNP) × SNP interactions (e.g. depression score × vitamin D interaction), and those with *P*-value annotations specifying non-European ancestry and/or non-additive models. Second, we identified the involvement of disease-associated genes in immunity using a custom collection of genes (‘ImmuneSet’)^20^ included in various curated immune gene sets from two of the main pathway databases, the immunity-specialised and expert-curated InnateDB,^21^ and the comprehensive human collection of the Molecular Signatures Database (MSigDB)^22^ containing canonical pathways and experimental signatures curated from publications. Both databases were accessed in August 2022. In total, 622 genes within the ImmuneSet associated with at least one of the phenotypes (unipolar depression, 236; type 2 diabetes, 377; obesity, 32; gout, 42) were selected for the study (Supplementary Table 1).

### DNAm data-sets

DNAm levels were measured in whole blood samples at three time points from the same participants using the Infinium MethylationEPIC BeadChip (Illumina Inc., San Diego, USA) array. Measurements took place at y0 and two consecutive follow-ups at 3-year intervals (y3 and y6). Here, three DNAm data-sets were created: one for the cross-sectional analysis at y0 and one for the longitudinal analysis of each diagnostic group (i.e. depression and control). All data-sets were processed in the same manner. Briefly, the raw intensity data, generated at Life&Brain GmbH (Bonn, Germany), were preprocessed, quality controlled and normalised using the R packages minfi^23^ and bigmelon^24^ following standard procedures. These included data conversion; calculation of detected *P*-values; removal of probes that (a) failed detection, (b) located on the sex chromosomes, (c) contained polymorphisms and/or (d) are cross-reactive; the removal of outlier samples (outlyx function of bigmelon); and calculation of normalised beta-values using the dasen method.^25^ Following the general recommendation,^26^ normalised betas were transformed to *M*-values for use in statistical analyses. Probes corresponding to the selected genes were identified using the array’s annotation file, in which probes are mapped to genes according to their genomic location. Because the effects of CpG sites (i.e. regions of DNA where a cytosine nucleotide is followed by a guanine nucleotide) located in gene bodies are less well known than those of CpG sites located near transcription start sites, only probes located within gene enhancer and promoter regions were considered here (i.e. sites in gene bodies were excluded). Therefore, the *M*-values of DNAm probes corresponding to enhancer/promoter regions of the selected disease-associated immune genes that passed quality control comprised the data-sets used for differential DNAm analyses. Individuals with missing data for the covariates (see below: Differential DNAm analyses) and those with fewer than three longitudinal measurements were excluded. The latter included individuals for whom at least one time point was identified as an outlier following assessment by principal component analysis using a threshold of mean ± 4 s.d. from the first dimension. Moreover, to create a control group, individuals with a lifetime diagnosis of depression up to y6 were removed from the population-based BiDirect cohort. Following quality control, 2747 probes corresponding to 382 of the selected genes were available for analysis; 276 and 274 depression cases, and 206 and 207 control individuals, remained for cross-sectional and longitudinal analyses, respectively (i.e. two case samples in the longitudinal analysis and one control sample in the cross-sectional analysis were excluded due to outlier behaviour during the respective quality control procedures).

### Cross-sectional differential DNAm analysis

DNAm levels at y0 were tested for differences between the depression (*n* = 276) and control (*n* = 206) groups. The analysis was performed with the limma^27^ R package. Because DNAm is susceptible to environmental and lifestyle factors,^28^ we considered the following to represent potential confounding factors in our sample: transitory differences in depressive symptom severity, including the presence of subclinical depressive symptoms in controls; disproportionate male/female ratios in the case and control groups; age differences; higher prevalence of obesity and diabetes in the depression group; and potential technical noise. Therefore, we adjusted our regression model for the following covariates: gender, age, weight, BMI, CES-D score, diabetes diagnosis and the first ten principal components of the DNAm data. Statistical significance was set to adjusted *P*-value (*q*) <0.05 and absolute log_2_-fold change (|log_FC_|) > 0.1. Exploratory significance was set to *P* < 0.05, with no threshold for |log_FC_| applied.

Case-control analysis was followed by gene set enrichment analysis using the GENE2FUNC tool from the FUMA-GWAS platform,^29^ where those genes annotated to DNAm probes with exploratory significance (exDMPs) were compared against a background list comprising the 4925 genes included in our complete ImmuneSet collection. Functional gene sets (i.e. lists of genes included in biological processes and pathways) and GWAS Catalog traits different from those related to type 2 diabetes, gout and markers of obesity, which were fundamentally enriched in our study, were considered over-represented at false discovery rate (FDR) < 0.05.

### Longitudinal differential DNAm analysis

As a second step, we performed follow-up longitudinal analyses of the set of exDMPs separately in the depression (*n* = 274) and control (*n* = 207) groups. To identify the presence of technical (batch) and other confounding effects, and to correct for these as necessary, we performed longitudinal assessments using the LongDat^30^ R package. LongDat screens for potential covariates on an individual variable basis and corrects for confounding effects as appropriate. This tool also provides information regarding dependency between the effects of the time variable and those of the covariates by generating a signal code, which can be either NS (non-significant effect), OK_nc (effect; no covariate), OK_nrc (effect non-reducible to covariate), OK_d (effect doubtful), EC (effect entangled with covariate) or RC (effect reducible to covariate). Although LongDat was originally developed for the analysis of microbiome data, the tool can be readily applied to other data modalities.^30^ Here, we tested for DNAm differences between the three time points measured in BiDirect (i.e. y0, y3 and y6) using the function for discrete time data (longdat_disc) applied to ‘others’ data type. As potential confounder variables we included the following: the array (sentrix identification) and slide (sentrix position) information from the MethylationEPIC sample sheet, gender, age, CES-D scores, weight, BMI, diabetes diagnosis and medications (i.e. number of antidepressants (ATC code N06A), number of other psychiatric medications (ATC code N05A), intake of medications for obesity or diabetes (ATC codes A08 and A10) and intake of immunomodulatory drugs (ATC codes L03 and L04)) at each time point, to account additionally for longitudinal changes in these features. The statistical significance for each comparison was defined at post hoc *q* < 0.05.

Longitudinal DNAm changes associated with depression were defined as probes that (a) changed significantly in at least one comparison following correction for covariates (if applicable) in the depression cohort; (b) showed effects that were independent from those of the covariates (if applicable) in cases (i.e. signal codes OK_nc and OK_nrc); and (c) were not significantly changed in controls following correction for covariates (if applicable; NS signal code) or showed effects that could be reduced to, or indiscernible only from, the effects of covariates in controls (signal code EC or RC). To provide a more integrative view of our findings, we classified the long-term change of each exDMP according to the observed longitudinal patterns as follows: (a) early: significant effect only at y3 (i.e. y0–3); (b) late: significant effect only at y6 (i.e. y0–6 with or without change in y3–6); (c) consistent: significant effects in all comparisons, where the end effect is in the same direction as that initially observed (i.e. y0–3 and y0–6 have the same direction of effect, even if the change in y3–6 is in the opposite direction); (d) progressive: significant effects in all comparisons, where the effect is in the same direction; (e) stable: the effect is observed at y3 and sustained thereafter (i.e. y0–3 and y0–6 have same direction of effect but there is no change in y3–6); (f) irrelevant: a significant effect in y3–6 was observed but not supported by the end effect (i.e. y0–6 showed no change); (g) neutral: opposing progressive changes nullify the end effect (i.e. y0–3 and y3–6 showed changes in opposing directions and there was no change in y0–6); (h) significant but non-specific: significant effect also observed in controls and with similar pattern; (i) inconclusive: effect indiscernible from that of covariates; and (j) NS: no significant effects in any comparison.

### Exploratory DNAm-based clustering analysis

Finally, we conducted an exploratory clustering analysis by applying an unsupervised clustering algorithm to the longitudinal DNAm data of exDMPs in the depression group, and tested for associations between cluster assignment and the baseline levels of various serum markers of inflammation. Statistical significance for these exploratory analyses was set to *P* < 0.05. Post hoc tests were conducted for findings below this threshold. Methods are described in detail in Supplementary Information.

## Results

**Fig. 1:**
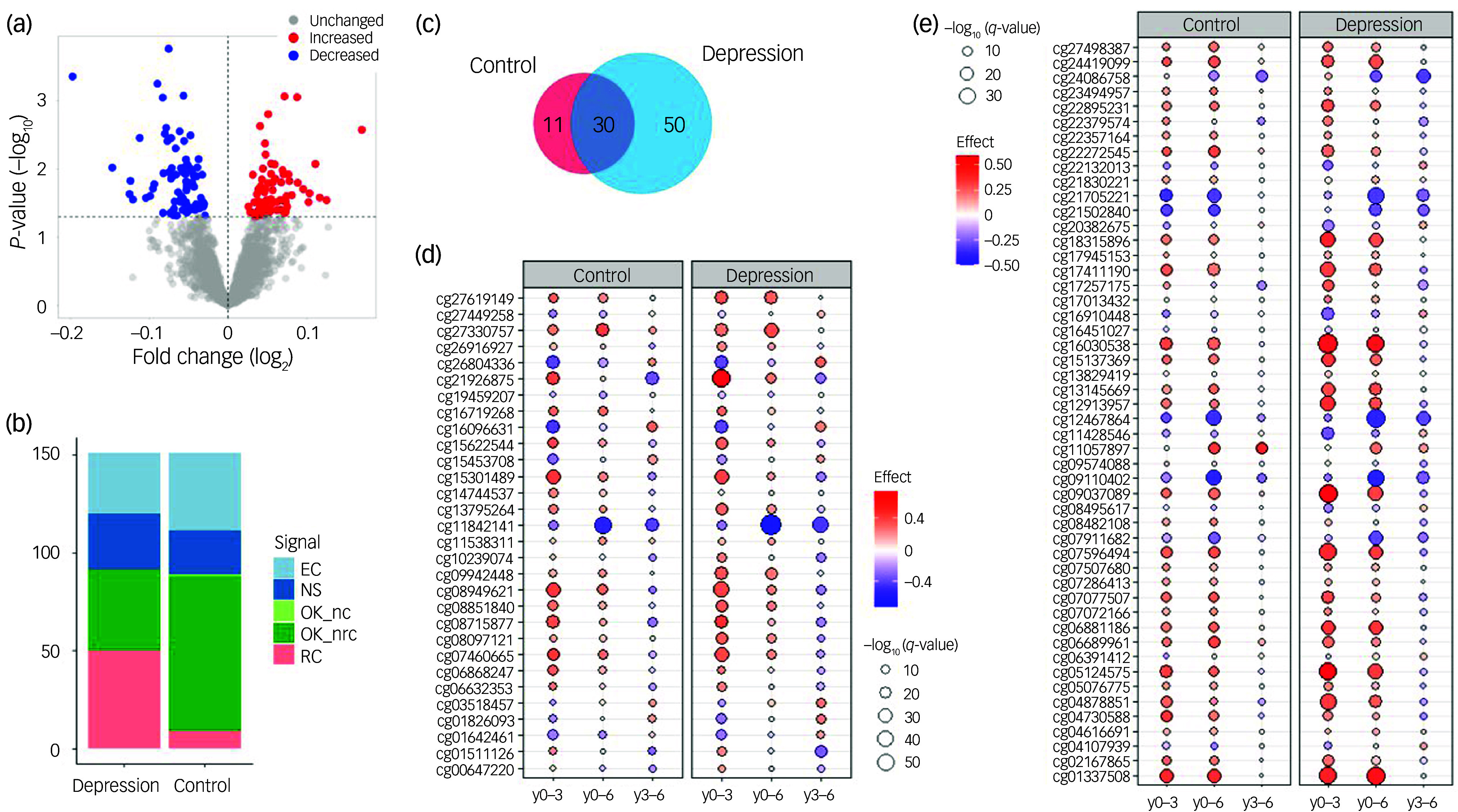
(a) Differential DNAm analysis between depression cases and controls. The volcano plot shows exDMPs, because no probes fulfilled the established criteria for statistical significance. Among 2747 probes tested, 75 were increased in cases while 76 were decreased under the exploratory threshold. (b) LongDat signal counts for longitudinal analyses in the depression and control cohorts. (c) Venn diagram of exDMPs with significant signals (i.e. OK_nc/nrc) in the longitudinal analyses in depression and controls. (d) Longitudinal changes in exDMPs that were significant in the depression and control cohorts. (e) Longitudinal changes in exDMPs that were significant in the depression but not in the control cohort. DNAm, DNA methylation; exDMPs, DNAm probes with exploratory significance; NS, non-significant effect; OK_nc, effect; no covariate; OK_nrc, effect non-reducible to covariate; OK_d, effect doubtful; EC, effect entangled with covariate; RC, effect reducible to covariate; y0–3, baseline to year 3; y0–6, baseline to year 6; y3–6, years 3–6.

**Fig. 2:**
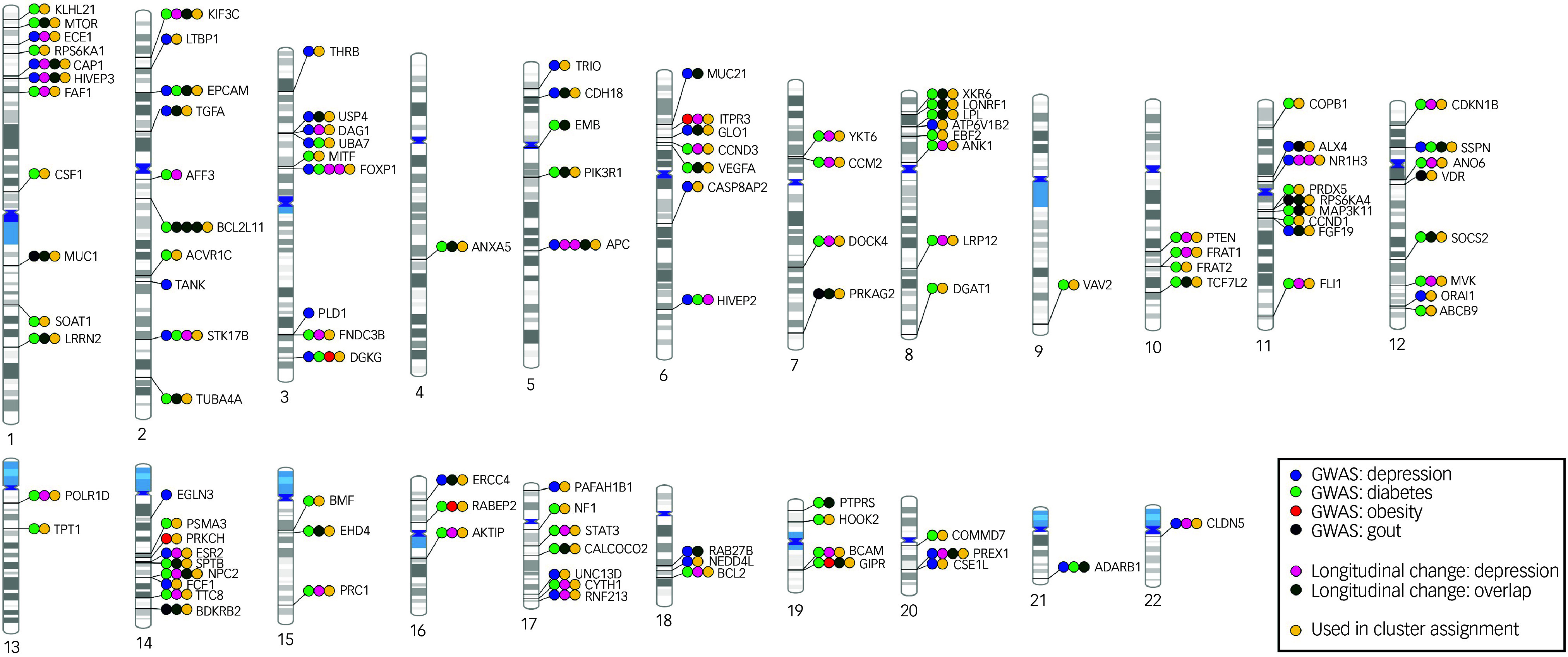
Phenogram summarising gene-level findings. The 113 genes corresponding to the 151 exDMPs identified in the case-control analysis at y0 are shown. exDMPs, DNAm probes with exploratory significance; y0, baseline; GWAS, Genome-Wide Association Study.

The basic characteristics of our longitudinal study sample are shown in Table 1. Cross-sectional analysis of differential DNAm between cases and controls at y0 found no DNAm probes that fulfilled the established criteria for statistical significance (*q* < 0.05 and |logFC|> 0.1). The lowest *P*-value was observed for the probe cg06881186 in the *BCL2* gene (log_FC_ = −0.075, *P* = 0.0002, *q* = 0.31). Therefore, we took the study further using the exploratory significance threshold (*P* < 0.05). With the latter, we found 151 exDMPs (Fig. 1(a) and Supplementary Table 2), among which 75 were increased in depression cases while 76 were decreased. These implicated 113 disease- and comorbidity-associated immune genes. Interestingly, a majority of exDMP genes (about 65%), including *BCL2*, are associated with diabetes in the GWAS Catalog (Fig. 2). Gene set enrichment analysis of the exDMPs compared against a background of immune-related genes showed over-representation (Supplementary Table 3) of signalling pathways that, in general, are involved in cell growth and proliferation, apoptosis and stress responses. In addition, we observed over-representation of genes associated with traits related to the numbers of white blood cells and, in particular, the numbers and morphological features of red blood cells, as well as blood lipid levels in the GWAS Catalog. Taken together, these results appear to suggest that exDMPs are enriched in genes generally involved in processes relevant to haematopoiesis.

**Table 1:** Basic description of the longitudinal BiDirect subsample used for this study

Parameters	Baseline		Year 3		Year 6	
	Cases	Controls	Cases	Controls	Cases	Controls
*n*	274	207	274	207	274	207
Females (%)	166 (60.6)	73 (35.3)	166 (60.6)	73 (35.3)	166 (60.6)	73 (35.3)
Age (mean ± s.d.)	51.3 (7.3)	52.5 (7.8)	54.3 (7.3)	55.5 (7.8)	57.7 (7.3)	58.5 (7.8)
CES-D (mean ± s.d.)	25.6 (12.2)	8.6 (6.5)	21 (12.4)	7.6 (6.3)	19.4 (11.6)	6.2 (5.3)
Body mass index (mean ± s.d.)	27.7 (5)	26.3 (3.8)	28.4 (5.1)	26.5 (4)	28.6 (5.4)	26.8 (4.1)
Weight (mean ± s.d.)	82.6 (17.6)	81.1 (13.9)	84.4 (17.8)	81.3 (14.3)	84.7 (18.4)	81.9 (14.4)
Diabetes (%)	14 (5.1)	4 (1.9)	21 (7.7)	7 (3.4)	31 (11.3)	8 (3.9)
Antidepressants (%)	230 (83.9)	4 (1.9)	187 (68.2)	3 (1.4)	163 (59.5)	3 (1.4)
More than 1 antidepressant (%)	35 (12.8)	1 (0.5)	26 (9.5)	0	21 (7.7)	0
Other psychiatric medications (%)	121 (44.2)	0	80 (29.2)	1 (0.5)	68 (24.8)	2 (1)
Obesity/diabetes medications (%)	14 (5.1)	2 (1)	17 (6.2)	4 (1.9)	18 (6.6)	5 (2.4)
Immunomodulators (%)	1 (0.4)	1 (0.5)	2 (0.7)	0	3 (1.1)	3 (1.4)

CES-D, Center for Epidemiological Studies-Depression.

Results of the longitudinal analyses of the 151 exDMPs in depression cases and controls can be found in Figs 1(b)–(e) and 2 and Supplementary Table 2. Overall, we observed that over half of the exDMPs (80, 53% from exDMPs) showed longitudinal changes that were independent from the effects of confounder variables related to technical, demographic, symptomatic, comorbidity and treatment features (i.e. OK_nc and OK_nrc signals) in the depression group (Fig. 1(b)). However, a number of these changes (30, 37.5% from significant changes) were also independently observed in the control group (Fig. 1(c) and (d)) and, therefore, longitudinal changes in only 50 exDMPs (33.1% from exDMPs, 62.5% from significant changes) were considered specific to depression in our study (Fig. 1(e)). For the 30 exDMP probes that changed in both groups, we mostly observed similar longitudinal patterns in depression and control individuals (Fig. 1(d)).

After careful manual integration of the results from our longitudinal analyses in depression cases and controls, we classified long-term changes in ten types according to the signals obtained for the depression and control groups, and the observed effects from independent time point comparisons (see Longitudinal differential DNAm analysis, above). Among the 50 exDMPs associated with depression, 7 probes showed patterns that could be considered neutral in the long term (Supplementary Table 2) and 1 showed a negative change in y3–6 that, due to the end effect being non-significant, was considered likely irrelevant. Therefore, among the 151 exDMPs explored longitudinally, we found that 42 associated with depression in the long term with the highest confidence (Table 2 and Fig. 2). Here, the type of long-term change patterns were classified as early (3), late (4), stable (15), consistent (15) and progressive (5). Longitudinal effects of age, depression severity, comorbidity and medication were broadly observed in these CpG sites (Table 2), but proved to be independent from the effects of the time variable in our analyses.

**Table 2:** Summary results of exDMPs with depression-associated longitudinal changes

Probe ID	Gene name	Chr	Gene GWAS association(s)	Change from controls	Long-term change	Change pattern	Other longitudinal effects (non-technical covariates)
cg17013432	*CAP1*	1	Depression	Down	Up	Stable	–
cg16910448	*ECE1*	1	Depression	Up	Down	Consistent	–
cg07911682	*FAF1*	1	Diabetes	Up	Down	Progressive	Age, CES-D, antidepressants
cg21830221	*HIVEP3*	1	Depression	Up	Up	Stable	–
cg11428546	*AFF3*	2	Diabetes	Up	Down	Consistent	Age
cg07596494	*KIF3C*	2	Diabetes	Down	Up	Consistent	Age, immunomodulators
cg22272545	*STK17B*	2	Depression, diabetes	Up	Up	Consistent	Age, diabetes
cg24086758	*DAG1*	3	Depression	Up	Down	Progressive (shifted)	Age, immunomodulators
cg08495617	*FNDC3B*	3	Diabetes	Down	Down	Consistent	–
cg20382675	*FOXP1*	3	Depression, diabetes	Up	Down	Consistent	Other psychiatric medications, obesity/diabetes medications
cg06391412	*FOXP1*	3	Depression, diabetes	Down	Up	Late	–
cg11057897	*APC*	5	Depression	Down	Up	Late	Age
cg18315896	*APC*	5	Depression	Down	Up	Stable	Age, diabetes
cg17945153	*CCND3*	6	Diabetes	Up	Up	Stable	BMI, CES-D, other psychiatric medications
cg13829419	*HIVEP2*	6	Depression, diabetes	Up	Up	Early	Age, immunomodulators
cg12913957	*ITPR3*	6	Obesity	Down	Up	Consistent	Age
cg07286413	*CCM2*	7	Diabetes	Up	Up	Stable	Immunomodulators, other psychiatric medications
cg07507680	*DOCK4*	7	Diabetes	Up	Up	Stable	CES-D, antidepressants, weight
cg16030538	*YKT6*	7	Diabetes	Down	Up	Consistent	Age, diabetes, immunomodulators
cg27498387	*ANK1*	8	Diabetes	Down	Up	Stable	Age, immunomodulators
cg15137369	*LRP12*	8	Diabetes	Down	Up	Stable	Age, weight
cg02167865	*FRAT1*	10	Diabetes	Up	Up	Consistent	Age, immunomodulators
cg04616691	*PTEN*	10	Diabetes	Up	Up	Late	–
cg09037089	*FLI1*	11	Diabetes	Down	Up	Consistent	Age, diabetes
cg05124575	*NR1H3*	11	Depression	Down	Up	Consistent	Age, diabetes, immunomodulators
cg24419099	*NR1H3*	11	Depression	Down	Up	Stable	Age
cg21705221	*ANO6*	12	Diabetes	Down	Down	Progressive	Age, diabetes
cg06689961	*CDKN1B*	12	Diabetes	Up	Up	Stable	Other psychiatric medications
cg21502840	*MVK*	12	Diabetes	Down	Down	Late	Age, CES-D
cg04730588	*POLR1D*	13	Diabetes	Up	Up	Consistent	–
cg23494957	*ESR2*	14	Depression	Up	Up	Early	–
cg05076775	*NPC2*	14	Diabetes	Up	Up	Stable	Age, CES-D, antidepressants
cg07077507	*TTC8*	14	Diabetes	Down	Up	Consistent	Age, other psychiatric medications
cg12467864	*PRC1*	15	Diabetes	Up	Down	Progressive	Age, BMI, diabetes
cg13145669	*AKTIP*	16	Diabetes	Down	Up	Stable	Age, diabetes
cg04878851	*CYTH1*	17	Diabetes	Down	Up	Consistent	Diabetes, obesity/diabetes medications
cg22357164	*RNF213*	17	Depression	Up	Up	Early	Age
cg01337508	*STAT3*	17	Diabetes	Up	Up	Stable	Age, diabetes, CES-D, antidepressants, obesity/diabetes medications
cg06881186	*BCL2*	18	Diabetes	Down	Up	Stable	Immunomodulators
cg22895231	*BCAM*	19	Diabetes	Down	Up	Stable	Age, other psychiatric medications, obesity/diabetes medications
cg09110402	*PREX1*	20	Depression	Down	Down	Progressive	Age, diabetes, CES-D, antidepressants
cg17411190	*CLDN5*	22	Depression	Up	Up	Consistent	Age, other psychiatric medications

exDMPs, DNAm probes with exploratory significance; chr, chromosome; GWAS, Genome-Wide Association Study; CES-D, Center for Epidemiological Studies-Depression; BMI, body mass index.

Finally, exploratory clustering analysis suggested three biological clusters of depression patients in BiDirect that did not correspond to the different time points in the data-set and appeared to present differential patterns of serum markers of inflammation at baseline. Among genes of relative importance in the analysis were *KIF3C*, *YKT6*, *NR1H3*, *FRAT1*, *ITPR3*, *FLI1* and *APC*. Full results of this exploratory analysis can be found in the Supplementary Information and Supplementary Table 4.

## Discussion

Depression and various medical conditions frequently coexist and are bidirectionally correlated.^19^ It is now believed that this relationship results from the presence of immune dysregulation, a common feature between depression and its major comorbidities.^6,7,10^ In this exploratory study, we investigated whether DNAm changes play a role in the immune dysregulation that contributes to depression and linked this with medical comorbidity (in this case, obesity, diabetes and gout) using a subsample from the German BiDirect Study. Cross-sectional analysis of a set of CpG sites regulating disease- and comorbidity-associated immune genes showed no differences between the depression and control groups. However, longitudinal analysis of the 151 top DNAm probes (exDMPs) found significant changes during a 6-year span in 42 CpG sites enriched in genes associated with diabetes, as well as traits and biological processes which, overall, fall into categories that might be relevant for haematopoiesis and mood regulation. Moreover, the longitudinal patterns of exDMPs identified three depression clusters that appeared to show differential profiles of serum markers of inflammation at baseline, supporting the existence of ‘immune’ depression subtypes. Overall, we show that BiDirect participants with depression at the time of recruitment had differential longitudinal patterns of DNAm in immune genes associated not only with depression but also with important comorbid conditions, particularly diabetes. These differential DNAm patterns appeared to relate to their baseline inflammatory profiles, and to be independent from the severity and course of depression, comorbidity status and effects of relevant medications, suggesting that the observed effects on DNAm might have resulted from the initial depression status in BiDirect participants. Based on our findings, we postulate that depression has long-term effects on DNAm in disease- and comorbidity-associated immune genes that have the potential to contribute to the link between depression and medical conditions. Therefore, we believe that our study advances understanding of immune dysregulation in depression and how this might link it with comorbidity, providing potential candidate genes for further investigations.

Case-control and quantitative (i.e. using scales measuring depression symptoms) analyses of DNAm performed in a genome-wide or candidate gene manner rendered a small number of significant associations in previous years, providing evidence of DNAm variation in genes such as *BDNF*, *COMT*, *TNNT3*, *CDC42BPB*, *ARHGEF3*, *KLK8* and *DAZAP2* being associated with depression.^31–35^ However, in epigenome-wide association studies (EWAS), such as that conducted by Li et al,^34^ exploratory-level findings showed enrichments in biological processes related to immunity and modest changes in immune genes, including *IL17RA* (interleukin-17 (IL-17) receptor A). More recently, the major depressive disorder (MDD) working group of the Psychiatric Genomics Consortium (PGC) conducted a large EWAS meta-analysis in 24 754 individuals from 18 international cohorts of European ancestry (including BiDirect).^36^ This study identified 15 CpG sites associated with depression, from which 5 were previously linked to immune-related traits and 2 to traits relevant to obesity. Importantly, the study also showed that depression-associated DNAm changes were correlated with the levels of inflammation markers in plasma, including IL-6, and that changes in the major histocompatibility complex (MHC) region have a causal relationship with depression. Our longitudinal study is therefore in agreement with previous reports, supporting the involvement of DNAm changes in the pathophysiology of depression and its links with comorbid medical conditions.

Because previous EWAS studies have already established that large samples are required to uncover statistically significant associations with depression, it is not surprising that our study found only modest cross-sectional differences. Although the BiDirect subsample used in our investigations is large for a longitudinal study, the total sample of 482 individuals proved to be underpowered for the cross-sectional study of our large set of candidate genes. It is possible that one of the reasons for this lies in the heterogeneity of immunological profiles in individuals with depression, which we uncovered through clustering analysis. Nevertheless, the strength of our study resides in the fact that it was designed to explore specifically immune DNAm dynamics as contributors to disease and medical comorbidity. To our knowledge, the latter has not previously been investigated. Moreover, the BiDirect Study provided a rare opportunity to assess this over the long term (i.e. 3 and 6 years after initial measurements). Various studies have investigated longitudinal changes in DNAm associated with the diagnosis of depression and/or its clinical presentation (e.g. disease duration and severity) in different contexts, including post-stroke depression,^37^ depression in breast cancer patients who underwent mastectomy^38^ and adolescents at risk of depression.^39^ However, these have been limited to either one candidate gene or a low number of individuals and follow-up DNAm measurements from a couple of weeks up to 1 year. Moreover, these have not investigated the links between depression and the underlying condition (when applicable).

Our study suggests that immune-related genes associated with the risk of depression and diabetes show small but statistically significant long-term changes in DNAm levels in subjects who experienced depression, regardless of disease severity and course, the presence of comorbidity or treatment with relevant medications. These time course changes in DNAm appear to correlate with the patterns of inflammation markers observed during the depression period. However, in this study we were not able to identify clinical features of depression or diabetes that correlated with the observed DNAm patterns and that might inform characteristics such as disease course or treatment response. Therefore, future investigations in this direction should be encouraged. In biological terms, our interpretation of gene set enrichment analysis using all protein-coding genes as background (data not shown, but summarised in Table 3) proposes haematopoietic imbalance, because we believe that the affected biological processes, which include cell growth, motility and death, as well as responses to hormones (including thyroid-stimulating hormone), might be related to the enriched haematopoietic traits, which included the counts and proportions of all blood cell populations. While it has been repeatedly observed that all, or specific populations of, white blood cells are altered in depressed individuals,^40^ reports of abnormalities of the entire haematopoietic compartment are scarce in the literature. Nevertheless, a general imbalance of haematopoiesis has previously been found in patients with major depressive episodes.^41^ Our hypothesis is further supported by observations in individuals undergoing haematopoietic stem cell transplantation. A considerable proportion of these patients (20–35%) develop depression, which has been shown to affect individuals’ inflammatory status and lead to further complications.^42^

**Table 3:** Summary of biological process and trait categories enriched in exDMPs with depression-associated longitudinal changes

Trait/process category	Source	Genes
Red blood cells	GWAS Catalog	*AFF3*, *STK17B*, *FOXP1*, *CCND3*, *ANK1*, *PTEN*, *FLI1*, *STAT3*, *BCL2*, *PREX1*, *FAF1*, *ESR2*
Platelets	GWAS Catalog	*AFF3*, *DAG1*, *CCND3*, *PTEN*, *FLI1*, *CDKN1B*, *BCL2*, *PREX1*
White blood cells	GWAS Catalog	*HIVEP3*, *STK17B*, *FOXP1*, *HIVEP2*, *CCM2*, *PTEN*, *FLI1*, *BCL2*, *FAF1*, *ANK1*, *NR1H3*, *PREX1*, *CCND3*, *MVK*, *FRAT1*
Mood instability	GWAS Catalog	*DAG1*, *HIVEP2*, *NR1H3*
Cell motility	GO_BP	*CAP1*, *DAG1*, *FOXP1*, *APC*, *DOCK4*, *LRP12*, *PTEN*, *ANO6*, *STAT3*, *BCL2*, *PREX1*, *CLDN5*, *ECE1*, *TTC8*
Growth and cell cycle regulation	GO_BP	*DAG1*, *FOXP1*, *CCM2*, *LRP12*, *PTEN*, *CDKN1B*, *ESR2*, *TTC8*, *STAT3*, *BCL2*, *APC*, *CCND3*
Cell death	GO_BP	*FAF1*, *STK17B*, *FOXP1*, *APC*, *PTEN*, *CDKN1B*, *ANO6*, *BCL2*
Response to hormone	GO_BP	*DAG1*, *FOXP1*, *APC*, *CCND3*, *NR1H3*, *CDKN1B*, *ESR2*, *STAT3*, *BCL2*

GWAS, Genome-Wide Association Study; GO_BP, Gene Ontology, biological process; exDMPs, DNAm probes with exploratory significance.

We acknowledge that our study has limitations – most importantly, the small sample size for cross-sectional analysis and the subsequently suboptimal statistical power. Sensitivity analyses excluding CES-D from and/or including medications in the model (data not shown) proved that the lack of statistically significant cross-sectional differences was not due to control bias. Because the probes selected for longitudinal analysis were based on top findings from an underpowered cross-sectional analysis, our observations should be interpreted with caution. Attempting to provide an interpretation of the types of longitudinal pattern of DNAm data was considered inadvisable given that other features, such as gene function, location of the CpG site, transcriptional programme and how the gene participates in disease processes, among others, would need to be considered, but an in-depth interpretation of changes in single genes lies beyond the scope of our manuscript. The interpretability, with respect to clinical implications of the results from our clustering approach, is limited by the lack of assessment of whether the different immune DNAm patient clusters showed differences in disease duration, treatment regimes or pharmacological responses. However, these investigations were outside the scope of the current work and will be addressed in future studies. In addition, the lack of transcriptional data for pairing with DNAm data could be seen as a limitation of our study, as this would allow us to corroborate whether the DNAm changes suggested by our study truly correlate with changes in the levels of expression of the corresponding genes. Nevertheless, the generation of transcriptomics data for this BiDirect subsample is under way and we would expect to be able to investigate this in the near future. Despite the current limitations, the results from our exploratory study are proof of concept of a role for immune regulation via longitudinal changes in DNAm profiles as a link between depression and medical comorbid conditions. This opens avenues for the investigation of novel therapeutic approaches to prevent and treat comorbidity in those with depression.

## Supporting information

Herrera-Rivero et al. supplementary material 1 Herrera-Rivero et al. supplementary material

Herrera-Rivero et al. supplementary material 2 Herrera-Rivero et al. supplementary material

## Data Availability

The data-sets that support the findings of this study are available from the first author (M.H.-R.) upon reasonable request.

## Supplementary material

The supplementary material can be found online at https://doi.org/10.1192/bjo.2025.10065
