# Supplementary material for: Immune DNA methylation in depression: cross-sectional and longitudinal study
Source: BJPsych Open. 2025 Jul 10;11(4):e129. doi: 10.1192/bjo.2025.10065 (PMC12247058; doi:10.1192/bjo.2025.10065)
Supplement: Herrera-Rivero et al. supplementary material 1 — Herrera-Rivero et al. supplementary material [file S2056472425100653sup001.pdf]

Immune DNA methylation in depression: A cross-sectional and longitudinal study

Marisol Herrera-Rivero, Matthias Nauck, Klaus Berger, Bernhard T. Baune

SUPPLEMENTARY INFORMATION

Exploratory DNAm-based clustering analysis

Methods

The Hierarchical Clustering based on Principal Components (HCPC) unsupervised clustering algorithm was applied to the longitudinal DNAm data (i.e. M-values for three time points) of exDMPs in the depression group using the FactoMineR [1] R package set to automatic detection of the appropriate number of clusters. We explored the contributions of individual exDMPs at the different time points to cluster assignment using the factoextra (https://rpkgs.datanovia.com/factoextra/index.html) R package. Then, we tested for associations between cluster assignment and the baseline log2-transformed levels of various serum markers of inflammation using analysis of covariance (ANCOVA) models adjusted for sex and baseline age, CES-D score, weight, BMI, diabetes diagnosis and medications. The panel of inflammation markers included: CRP, macrophage inflammatory protein-1 (MIP-1) alpha (CCL3) and beta (CCL4), interleukins (IL) -1beta, -4, -6, -12p70 and -17a, interferon gamma (IFN-g), tumor necrosis factor alpha (TNF-a) and intracellular adhesion molecule-1 (ICAM-1). We also tested for associations of each covariate with the DNAm clusters while adjusting for the rest of covariates in the model to assess whether the identified clusters might correspond to any of these clinical features. The suggested significance for these models was set to $p<0.05$. When the ANCOVA result was below this threshold, we carried out a *post-hoc* analysis using the TukeyHSD test, considering statistical significance for the latter at $q<0.05$. These statistical analyses were performed with R.

Results

HCPC identified three biological clusters of depression patients (Figure S1A), here c1 (n=93), c2 (n=92) and c3 (n=89). Within the variables used by the algorithm for assignment to each cluster, we observed that all three time points for 14 exDMPs were significant contributors to all clusters (Figure S1B) and at least half of these were within our curated set of depression-associated exDMPs with long-term changes. The latter corresponded to the genes *KIF3C*, *YKT6*, *NR1H3, FRAT1*, *ITPR3*, *FLI1* and *APC*. All contributions of exDMPs per time point to the assignment of DNAm clusters can be found in Suppl.Table.4. Because all three time points of most exDMPs contributed in more or less extent to cluster assignment, the identified clusters did not correspond to the different time points in the dataset. Moreover, we found evidence suggesting that these DNAm clusters might present different patterns of serum markers of inflammation at baseline

(Figure S1C). Here, we found differences in the (log2) y0 serum levels of IL-17a (q=0.0035) between c1 and c2, and of ICAM-1 ($q=2.8x10^{-4}$), IFN-g (q=0.0103), IL-6 (q=0.0148), MIP-1a (q=0.03) and MIP-1b (q=0.037) between c1 and c3 in the *post-hoc* analyses. The individual markers showed some degree of confounding effects ($p<0.05$) according to our regression models. For example, we found indication that ICAM-1 levels were affected by age, as those of IFN-g by sex and MIP-1b by BMI, while levels of IL-17a were affected by both sex and BMI, and those of MIP-1a and IL-6 by treatment with antidepressants and obesity/diabetes medications, respectively. However, these confounding features were not representative of the patient DNAm clusters, as we found no association between clusters and age, sex, CES-D score, weight, BMI, diabetes or medications.

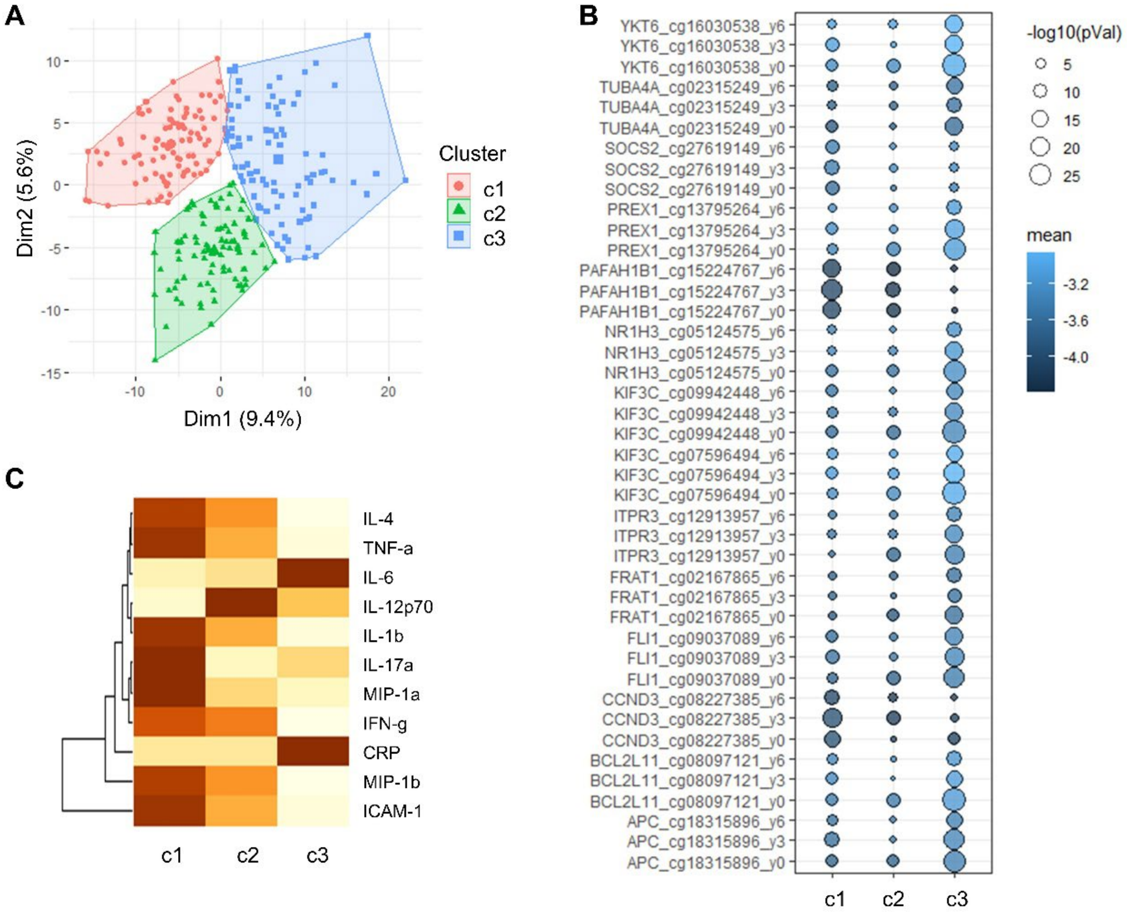

Figure S1. A) HCPC clustering identified three biological clusters of cases based on longitudinal DNAm data for the exDMPs (individuals in clusters: c1=93, c2=92, c3=89). **B)** The exDMPs contributing to assignment to all DNAm cluster by all three time points are shown. The cluster mean and p-values of the three time points are represented by circle colors and sizes, respectively. **C)** The DNAm clusters showed different patterns of serum markers of inflammation. Here, mean values scaled by rows are presented.

References

1. Lê S, Josse J, Husson F. FactoMineR: An R Package for Multivariate Analysis. Journal of Statistical Software. 2008;25(1):1-18.
